# Mediastinal Intrathymic Symptomatic Parathyroid Adenoma and Robot-Assisted Thoracoscopic Surgery (RATS) Approach

**DOI:** 10.7759/cureus.96426

**Published:** 2025-11-09

**Authors:** Jason Calderon Sanchez, Pablo Gomes-da Silva de Rosenzweig, Mostafa Ahmed, Luis-Angel Hernandez-Arenas

**Affiliations:** 1 Cardiothoracic Surgery, University Hospitals Coventry and Warwickshire, Coventry, GBR; 2 Experimental Lung Transplant Unit, Instituto Nacional de Enfermedades Respiratorias, Mexico City, MEX

**Keywords:** ectopic parathyroid, hyperparathyroidism, mediastinal lesions, primary hyperparathyroidism, rats

## Abstract

Anterior mediastinal masses, including thymomas, lymphomas, and ectopic parathyroid adenomas, pose diagnostic and therapeutic challenges due to anatomical complexity and diverse aetiologies. Symptoms often stem from local compression, requiring multimodal imaging such as CT, MRI, and PET for accurate diagnosis. Primary hyperparathyroidism, frequently linked to ectopic parathyroid adenomas, causes hypercalcaemia and systemic complications. Traditionally managed via sternotomy, advancements in minimally invasive techniques, particularly video-assisted (VATS) and robot-assisted thoracoscopic surgeries (RATS), have improved outcomes. This report highlights a 50-year-old male patient with hyperparathyroidism and hypercalcaemia who underwent successful RATS for a 2.9 cm anterior mediastinal mass. Postoperatively, his calcium and parathyroid hormone levels normalised, demonstrating RATS as a precise and effective option for complex mediastinal lesions.

## Introduction

Primary hyperparathyroidism (PHP) is the most common cause of hypercalcaemia in the outpatient setting and is characterised by autonomous secretion of parathyroid hormone (PTH), leading to renal, skeletal, and neuropsychiatric complications [1,2]. While many patients are diagnosed incidentally, symptomatic disease may present with nephrolithiasis, bone pain, fragility fractures, muscle weakness, or skeletal deformities [2]. Neuropsychiatric symptoms such as fatigue, depression, anxiety, and cognitive dysfunction are also reported, affecting up to 23% of patients [3].

The aetiology of PHP is heterogeneous: approximately 80% of cases are caused by a solitary adenoma, 15% by multiglandular hyperplasia, 2-4% by multiple adenomas, and fewer than 1% by parathyroid carcinoma [4]. According to the World Health Organization (WHO) Classification of Endocrine and Neuroendocrine Tumours (2022), parathyroid neoplasms are classified as adenomas, atypical adenomas, and carcinomas, with carcinoma representing fewer than 1% of cases but an important differential [4]. Familial syndromes, including multiple endocrine neoplasia (MEN), hyperparathyroidism-jaw tumour syndrome, and isolated familial hyperparathyroidism, account for approximately 5% of presentations [5,6].

Diagnosis of PHP requires the presence of hypercalcaemia with an elevated or inappropriately normal PTH level [7]. Confirmation of true hypercalcaemia is essential and requires either correction for serum albumin or measurement of ionised calcium [8]. Imaging is performed for localisation prior to surgery. First-line modalities are neck ultrasound and technetium-99m sestamibi scintigraphy, although sestamibi may be negative in ectopic glands due to the small adenoma size, low oxyphil cell content, or mediastinal location [9].

Ectopic parathyroid glands are reported in up to 22% of cases [9]. The most frequent ectopic locations include the thymus (38%), retro-paraesophageal region (31%), intrathyroidal tissue (18%), mediastinum (6%), undescended along the embryonic tract (4%), and the carotid sheath (3%) [9]. Such atypical locations are clinically significant, as intrathymic or mediastinal adenomas may appear radiographically as anterior mediastinal masses.

Anterior mediastinal masses include a broad differential, most classically thymoma, teratoma, thyroid tissue, and lymphoma (“the 4Ts”), but ectopic parathyroid adenomas are an important and often overlooked consideration [10,11]. Historically, mediastinal parathyroid adenomas were excised via median sternotomy [12]. More recently, minimally invasive techniques such as video-assisted thoracoscopic surgery (VATS) and robot-assisted thoracoscopic surgery (RATS) have allowed for improved visualisation and precision with reduced morbidity compared to open approaches [13,14].

This report aims to highlight the diagnostic challenge of intrathymic parathyroid adenomas presenting as mediastinal lesions with negative sestamibi uptake and illustrate the role of minimally invasive thoracic surgery in the management of intrathymic parathyroid disease.

## Case presentation

A 50-year-old male patient presented to his general physician (GP) with severe bone pain, lethargy, poor concentration, and fluctuating mood lasting eight weeks, impairing his ability to work. Blood tests revealed elevated serum calcium (3.11 mmol/L), alkaline phosphatase (620 U/L), and PTH (87.1 pmol/L), with reduced phosphate (0.54 mmol/L), suggesting PHP with evidence of skeletal involvement (Table 1).

**Table 1:** Laboratory findings before surgery in a patient with primary hyperparathyroidism

Parameter	Preoperative value (SI Units)	Preoperative value (conventional units)	Reference range (SI units)	Reference range (conventional units)
Serum calcium	3.11 mmol/L	12.4 mg/dL	2.10-2.60 mmol/L	8.5-10.2 mg/dL
Phosphate (serum)	0.54 mmol/L	1.67 mg/dL	0.80-1.50 mmol/L	2.5-4.5 mg/dL
Alkaline phosphatase (ALP)	620 U/L	-	44-147 U/L	-
Parathyroid hormone (PTH)	87.1 pmol/L	820 pg/mL	1.6-6.9 pmol/L	15-65 pg/mL

**Figure 1:**
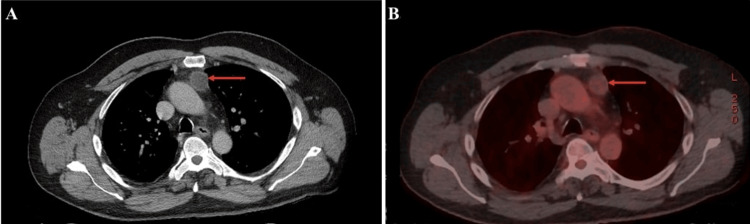
Preoperative (a) CT and (b) PET-CT showing a 2.9 cm low attenuating nodule with some peripheral enhancement and mild adjacent fat stranding within the anterior mediastinal fat just adjacent the ascending aorta

Referred for further evaluation, a neck ultrasound showed no abnormalities. A technetium-99m sestamibi scan demonstrated no abnormal uptake in the neck to suggest parathyroid pathology. However, the same scan incidentally revealed a mediastinal lesion that was visualised anatomically but showed no radioisotope retention and no associated lymphadenopathy. Following this finding, a PET-CT scan was carried out, which confirmed the presence of an anterior mediastinal mass (Figure 1). 

**Figure 2:**
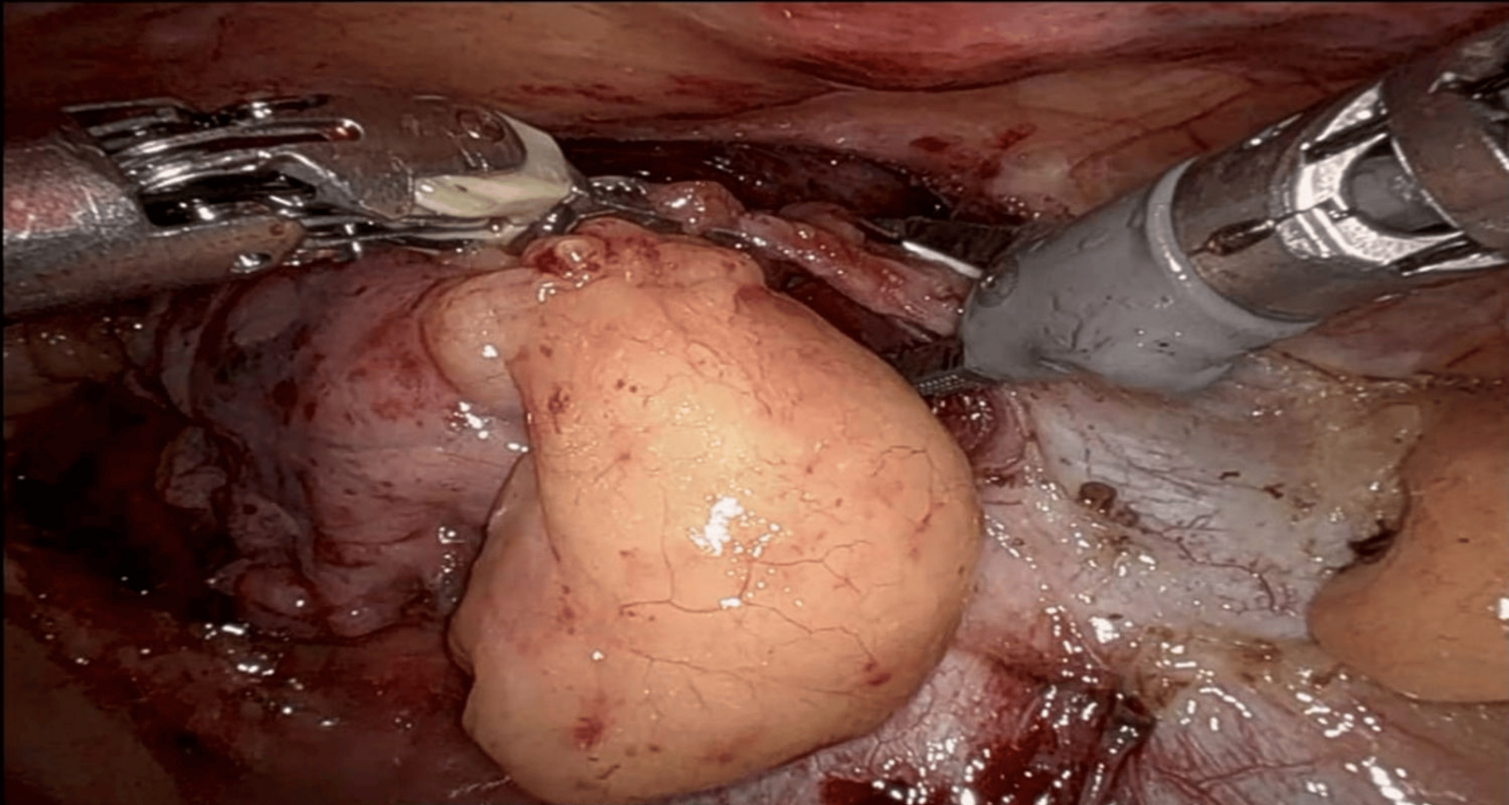
Intraoperative image of the intrathymic tumour resected through the left anterior three-port RATS RATS: robot-assisted thoracoscopic surgery

The case was reviewed at a multidisciplinary meeting, where an ectopic parathyroid adenoma was suspected. Referred to thoracic surgery, the patient underwent RATS thymectomy, with no complications (Figure 2).

**Figure 3:**
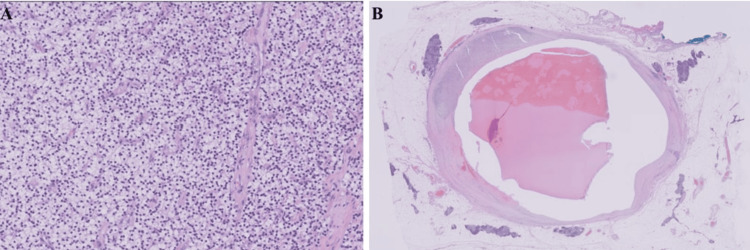
Postoperative histology findings. (a) H&E stain at 20X showing solid sheets of water clear cells; (b) H&E stain at 0.5x magnification. This shows an intrathymic cystic H&E: hematoxylin and eosin

Pathology revealed a cystic intrathymic parathyroid adenoma surrounded by normal thymic tissue (Figure 3).

Postoperatively, calcium levels (2.20 mmol/L) and PTH (48 pg/mL) both normalised (Table 2).

**Table 2:** Laboratory findings before and after surgery in a patient with primary hyperparathyroidism

Parameter	Preoperative value (SI units)	Preoperative value (conventional units)	Postoperative value (SI units)	Postoperative value (conventional units)	Reference range (SI units)	Reference range (conventional units)
Serum calcium	3.11 mmol/L	12.4 mg/dL	2.20 mmol/L	8.81 mg/dL	2.10-2.60 mmol/L	8.5-10.2 mg/dL
Parathyroid hormone (PTH)	87.1 pmol/L	820 pg/mL	5.1 pmol/L	48 pg/mL	1.6-6.9 pmol/L	15-65 pg/mL

The patient showed clinical improvement with no postoperative complications and was discharged on the third postoperative day. On follow-up at eight weeks, the patient was asymptomatic and recovered from the surgical procedure.

## Discussion

Anterior mediastinal masses comprise a diverse group of lesions, including both neoplastic and non‑neoplastic types, often requiring a multidisciplinary approach due to their complex aetiologies and proximity to vital structures [1]. Occupying the anterior compartment in 50% of cases, common lesions include the 4Ts: thymomas, teratomas, thyroid tissue, and lymphomas. Thymic malignancies account for 35% of cases, followed by lymphomas at 25% (13% Hodgkin’s lymphoma and 12% non‑Hodgkin’s lymphoma). Less common causes include thyroid or endocrine tumours (15%), benign teratomas (10%), and malignant germ cell tumours (10%), with seminomas comprising 4% and non‑seminomatous germ cell tumours 7% [1-2]. Rarely, an anterior mediastinal mass may arise from ectopic parathyroid tissue, complicating differential diagnoses [3].

Ectopic parathyroid adenomas occur in up to 22% of cases, typically due to abnormal embryologic migration. Inferior parathyroid glands are more prone to ectopy due to their prolonged descent during development. Superior glands are commonly located in the tracheoesophageal groove (45%) or posterior mediastinum (15%) [6].

PHP, often caused by a single adenoma (80-85% of cases), is characterised by excessive PTH secretion, leading to hypercalcaemia and complications such as nephrolithiasis, skeletal fragility, and neuropsychiatric symptoms [7-8]. While some patients are asymptomatic, others present with bone pain, fractures, muscle weakness, or mood disturbances [8]. Less common causes of PHP include hyperplasia of all four glands (15%), multiple adenomas (2-4%), or parathyroid carcinoma (<1%) [7].

Localisation of ectopic adenomas is crucial for effective management. The gold standard remains single‑radioisotope scintigraphy with technetium‑99m combined with single‑photon emission computed tomography (SPECT), providing 91-98% sensitivity [7]. Traditionally, bilateral four‑gland exploration was the preferred surgical approach. However, minimally invasive parathyroidectomy has largely replaced it for single adenomas, improving outcomes [9]. Ectopic glands in the upper mediastinum can sometimes be accessed via a cervical incision, but deeper glands, such as those in the thymus, often necessitate a thoracic approach [4].

The historical gold standard for thymectomy has been median sternotomy, an invasive procedure involving a long bone incision, associated with risks such as bleeding, infection, and postoperative pain [10]. Advances in minimally invasive techniques, including partial sternotomy, transcervical approaches, and VATS, have significantly reduced these complications. Among these, RATS has emerged as a particularly advantageous method due to its superior three‑dimensional visualisation, greater dexterity, and tremor‑free precision enabled by the da Vinci robotic system [5].

Clinical reports demonstrate the evolving role of minimally invasive techniques in managing mediastinal pathology. Akin et al. and Gurluler et al. reported successful VATS cases for ectopic mediastinal parathyroid adenomas [11-12]. A systematic review by Wang et al. further highlighted the advantages of RATS over sternotomy for thymectomy, citing reduced complications and enhanced precision [13]. Similarly, Zhu et al. analysed 113 patients with thymic tumours, concluding that RATS offers superior outcomes, especially for larger tumours requiring complex resections [14].

## Conclusions

The surgical management of anterior mediastinal masses remains challenging due to their anatomical complexity and proximity to critical structures. Although traditional sternotomy has been the standard for decades, it is associated with significant risks, including postoperative pain, infection, and prolonged recovery. Minimally invasive techniques such as VATS and RATS have redefined surgical management, offering safer and more effective solutions. VATS, widely adopted for thymectomies, minimises recovery time and complications, while RATS further enhances outcomes with improved visualisation, dexterity, and precision. RATS appears to be particularly beneficial for complex resections, such as those involving large thymic tumours or ectopic parathyroid adenomas, and has emerged as a compelling method in select cases. These advancements in surgical techniques continue to improve safety, efficacy, and patient outcomes in managing challenging mediastinal pathology.
